# The Dynamic Target Motion Perception Mechanism of Tactile-Assisted Vision in MR Environments

**DOI:** 10.3390/s22228931

**Published:** 2022-11-18

**Authors:** Wei Wang, Ning Xu, Haiping Liu, Jue Qu, Sina Dang, Xuefeng Hong

**Affiliations:** Air Defense and Antimissile School, Air Force Engineering University, Xi’an 710051, China

**Keywords:** mixed reality, motion perception, tactile assistance, tactile sensor, velocity and position, cognitive mechanisms

## Abstract

In the mixed reality (MR) environment, the task of target motion perception is usually undertaken by vision. This approach suffers from poor discrimination and high cognitive load when the tasks are complex. This cannot meet the needs of the air traffic control field for rapid capture and precise positioning of the dynamic targets in the air. Based on this problem, we conducted a multimodal optimization study on target motion perception judgment by controlling the hand tactile sensor to achieve the use of tactile sensation to assist vision in MR environment. This allows it to adapt to the requirements of future development-led interactive tasks under the mixed reality holographic aviation tower. Motion perception tasks are usually divided into urgency sensing for multiple targets and precise position tracking for single targets according to the number of targets and task division. Therefore, in this paper, we designed experiments to investigate the correlation between tactile intensity-velocity correspondence and target urgency, and the correlation between the PRS (position, rhythm, sequence) tactile indication scheme and position tracking. We also evaluated it through comprehensive experiment. We obtained the following conclusions: (1) high, higher, medium, lower, and low tactile intensities would bias human visual cognitive induction to fast, faster, medium, slower, and slow motion targets. Additionally, this correspondence can significantly improve the efficiency of the participants’ judgment of target urgency; (2) under the PRS tactile indication scheme, position-based rhythm and sequence cues can improve the judgment effect of human tracking target dynamic position, and the effect of adding rhythm cues is better. However, when adding rhythm and sequence cues at the same time, it can cause clutter; (3) tactile assisted vision has a good improvement effect on the comprehensive perception of dynamic target movement. The above findings are useful for the study of target motion perception in MR environments and provide a theoretical basis for subsequent research on the cognitive mechanism and quantitative of tactile indication in MR environment.

## 1. Introduction

Currently, operators in aviation towers perform control and real-time scheduling of tasks for velocity and position sensing of air targets mainly through dynamic channel interfaces [1], which have an important role in air traffic, aerospace engineering, aviation military, and other fields [2,3,4]. The existing dynamic channel control interface consists mainly of a linear motion approach display with graduated line segments, target object cursors, decision point cursors, and related information. The relative motion of the target position point and the decision point is used to show the relative position relationship between the target and the decision point, and when the position cursor representing the target coincides with the decision point, the operator can start the decision. As an important human–computer interaction interface to characterize the target motion condition, the dynamic channel interface has dynamic and intuitive characteristics [5]. It brings very good interaction effects to trajectory determination, target tracking, multi-target motion perception [6,7,8], and other types of tasks, as well as improving task effectiveness. Additionally, due to the complexity of the real situation and the diversity of system elements [9], it is usually difficult for a single dynamic channel to describe the overall motion, and multiple parallel channels are needed for simultaneous sensing and decision-making [10]. Therefore, there are two types of dynamic process tasks, a multi-process simultaneous sensing task [11] and a single-process dynamic accurate tracking task [12]. This corresponds to two scenarios of multi-target and single-target tasks. In recent years, holographic tower control based on virtual reality and mixed reality devices is entering the daily work of aviation practitioners, and its advantages of easy interaction, large amount of information and full immersion show a good future development trend. As the elements in the mixed reality field of view become more and more, the dimensionality of information is increasing, the pressure of human visual cognition is increasing, and its perception of motion and tracking of the position will become less effective. Multimodal-assisted human–computer interaction is a method of adding other sensory feedback methods to assist visual interaction. It can improve the overall interaction effect and efficiency without adding the burden of visual fatigue [13]. Studies have shown that tactile has a good cueing and warning effect for situations such as navigation and perceived object movement [14,15,16]. Therefore, the use of tactile assistance to cue visual perception of motion is effective. In this paper, tactile feedback is incorporated in a mixed reality environment to assist the operator in multi-target decision making and accurate tracking. The tactile intensity and different types of tactile schemes are combined with the perception of velocity and position to assist the motion characteristics of targets in MR 3D environments.

The intensity of the sense of touch has an important effect on the human perception of target motion. Andrew A. Stanley [17] showed that tactile cues can guide users through different intensity tactile vibration cues from wearable tactile devices to guide velocity-based motion processes, such as position localization and trajectory tracking. Experiments by I. Hachen [18] found that applying different vibration stimuli to a person’s fingertips caused a bias in the judgment of average velocity, and the value of the bias increased between experimental groups as the duration of the experiment increased. Chris J. Dallmann [19] determined tactile -based velocity changes by hypothesizing a combination of vibration induced motion and other tactile vibrations. The discrimination of vibration induced and tactile vibration velocities was derived from sensor measurements. Alison I. Weber [20] simulated a human touching an object by using a stimulator to scan the human finger for stimulation, and when the scanning speed changed, the tactile sensation then changed, and the human perceived speed changed as well with its effect on neurons, which also concerned temporal and spatial coding. Motoki Tachiiri [21] investigated the boundary model of velocity change based on velocity and acceleration due to haptics and the effect of human psychological response to that velocity perception, and the results showed that haptics can lead to bias in human perception of velocity. 

The variation of the tactile vibration modality is an important characteristic to distinguish the different tactile instructions. The variation of its mode depends on three elements: vibration position, vibration rhythm, and vibration sequence. They also constitute the vibration scheme of PRS (position, rhythm, vibration) [22]. They also have important applications in motion, process scheduling, and decision-making judgments [23]. The PRS indication scheme for tactile cues is used as an effective auxiliary indication scheme for precise description and analysis of tactile. Alexandra List [24] noted that changes in tactile location interfere with human visual judgments of target objects, but that overall tactile feedback based on tactile location significantly contributes to the perceptual efficiency of vision, which reflects the emergence of a visual-touch cross-modal relationship. In-Seon Lee [25] showed that the brain’s response to tactile stimuli would enable a person to distinguish the location of tactile stimuli on the body surface, and quantified the coding of said areas. Based on this principle, he also investigated the efficiency of human use of touch to perceive spatial location, and obtained more optimistic conclusions. Verena N. Buchholz [26] analyzed the response of the human brain to positional information in a spatial coordinate system after adding fingertip stimuli of different rhythms, concluding that the cognitive induction effect of the brain is enhanced by simultaneous visual-tactile stimulation and that the efficiency of human perception of spatial representations increases. Jakob Voigts [27] concluded the important role of tactile rhythm in discriminating the position of objects by studying the different vibration rhythms caused by the tremulous movements of the mouse whiskers in contact with objects. Wenbo Huang [28] adopted a PRS vibration scheme to identify the pilot’s flight attitude, and the vibration position, rhythm, and sequence played a key role in prompting flight crew information, which also significantly improved the interaction efficiency. The work of Assumpcao [29] investigated the reference system behind tactile contextual memory, in which different sequences of vibrations have different effects on manual gesture control and learning, and in which human spatial attention was found to be associated with relevant tactile cues to enhance learning efficiency. 

In summary, it is shown that among visual and tactile cross-modal interactions, there is a strong correlation between tactile intensity and target velocity, and tactile indication scheme and target location acquisition. It is also widely used in the fields of transportation, aerospace, medicine, and geographic navigation. However, there is still a gap in the research of tactile-assisted visual perception of target motion in MR environments, and it is doubtful whether the above mechanism in the real state can be well adapted and applied in MR environments. This leaves a gap in the need for efficient perception of target velocity, position, and other motion characteristics in the MR environment, and also imposes constraints on the spatial perception in the holographic state. Therefore, this paper designs experiments to investigate the mapping between tactile intensity and velocity, the PRS vibration indication scheme and precise position localization, and the experiments are designed to verify the effect.

## 2. Experimental Procedures and Methods

### 2.1. Experimental Procedure

This section is divided into two experiments. The first experiment is a target importance discrimination experiment with multiple dynamic process channels, and the second experiment is a precise position control experiment with a single process channel. They verify the effect of tactile-assisted vision for dynamic processes in MR environment, respectively.

Subjects: 30 master’s students from an engineering university (age range 23–35, mean 25.6, SD = 2.67), all subjects had some experience in mixed reality manipulation, had no limb residuals in the finger area, and had normal visual and tactile senses. They were not informed of the purpose of the study and participated in the experiment with informed consent.

**Figure 1:**
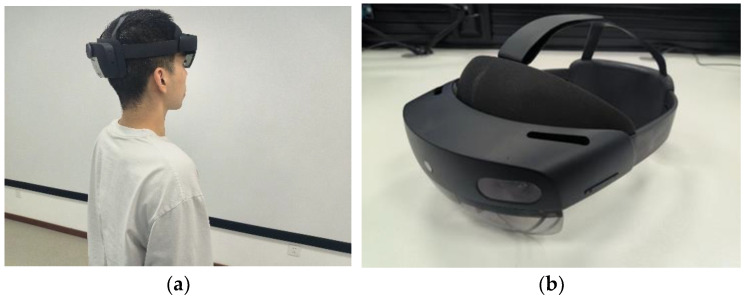
Visual presentation equipment and experimental scene: (**a**) subjects wearing the device; (**b**) Hololens2 display device.

Equipment and target materials: The experimental scenario was presented in HoloLens2 generation, an MR device that takes a transparent holographic lens to achieve a 3D display based on eye position, and with six cameras and depth sensors to support holographic interaction scenarios such as line of sight tracking, touch, and grip. Participants wear the device on their heads and stand in a relatively empty room with a certain amount of visible light, the scene and equipment are shown in Figure 1.

**Figure 2:**
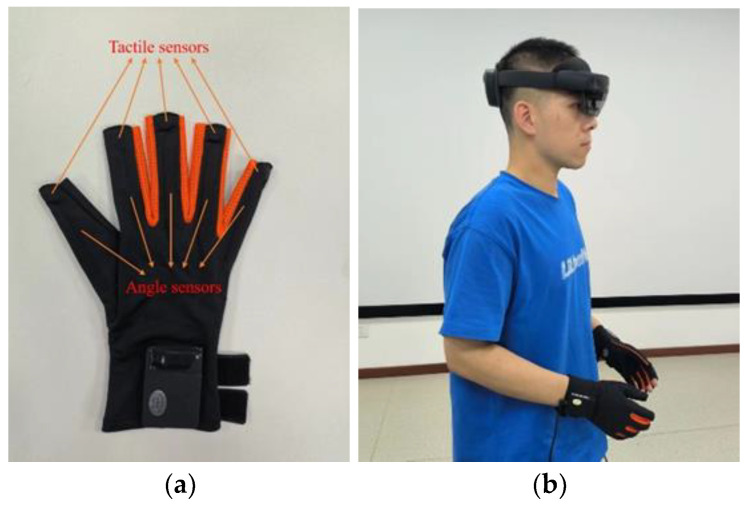
Tactile feedback equipment and experimental scene: (**a**) tactile feedback devices; (**b**) subject wearing the device.

Hand-based tactile feedback is implemented in the following form: a tactile glove with tactile sensors on each finger (Figure 2). The glove is equipped with tactile sensors at each fingertip (5 in total) to achieve tactile force feedback. In the middle part of each finger, the angle fiber optic sensors (5 in total) are equipped to record the angle of the finger bending with dynamic and static accuracy of 0.2 degrees and 0.02 degrees, respectively. The data update rate of the above sensors is 100 hz, and the USB interface is adopted to connect to the main device, and its power supply is 5 v @ 300 mA.

The scene of the overall experiment was edited by Unity3D software. After corresponding the COM port numbers in the script one by one and setting the data baud rate to 256,000, the vibration of each finger tactile sensor of the glove can be driven in the embedded script in Unity. Additionally, the specific vibration intensity and interval time can be set in Visual Studio, which meets the requirements of this paper for the principles and experiments of vibration-assisted vision research2.1.1 Data analysis of multi-target indication.

#### 2.1.1. Experimental Procedure of Multi-Target Indication

**Figure 3:**
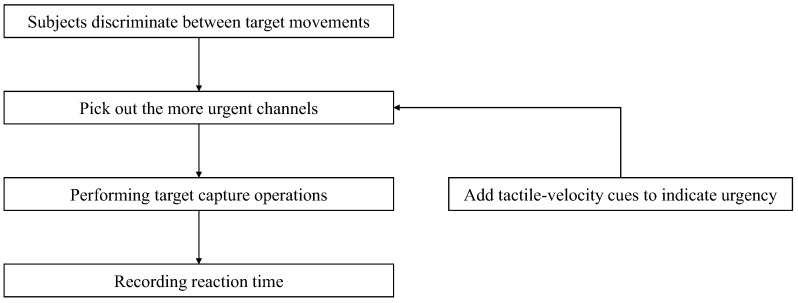
Process of Experiment 1.

This experiment intends to simulate the operation related to the dynamic channel interface of a holographic aviation tower. It is geared towards the task of judging the urgency of multiple objectives. In this paper, the urgency refers to the time for the target to reach the specified position. Since the target movement speed and distance of each dynamic process are different, when the arrival time is similar, redundancy and misjudgment will occur when relying solely on vision to judge the expected time. By setting up dynamic processes with different intensity stimuli corresponding to different speeds, the correspondence between different tactile intensities and different speed movements was investigated. After obtaining this correspondence, the tactile indication is then used instead of speed to indicate the corresponding target of the most urgent passage at a similar degree of urgency. The specific flow of the experiment is shown in Figure 3.

In the first stage of this experiment, the relationship between tactile intensity and velocity was first studied: the starting and ending points of the target motion in each channel were set to be the same, and their arrival times differed due to different target velocities, which were arranged in the following sequence in Table 1.

According to [30], the most sensitive interval for the human to perceive tactile intensity is 0–4 v, so this paper envisages that equalizing the intensity by range within this interval can make subjects perceive different intensities, and fortunately, this idea was proved in Section 2.2. Therefore, the tactile intensity of the experiment was accordingly divided into 5 steps in the actual range 0–4 V. The adjustment of the intensity in the VS is implemented as an array. Sensor values from 0–252 for no vibration to maximum vibration intensity. Sensor values of 32, 87, 142, 197, and 252 were chosen to represent low, lower, medium, higher, and high-intensity tactile stimuli, which is shown in Table 2.

Before starting the experiment, we first told the subjects that tactile intensity and speed were correlated. The subjects were then informed to select the channel within the visual field that matched the speed of the intensity after perceiving the intensity. In other words, we applied the above-mentioned intensity levels (low, lower, medium, higher, and high) to the thumb position of the subject’s gloved hand. The subject then visually observed the different movement speeds of the five channels and selected the corresponding speed of the channel that could be perceived and then performed a “finger click” on the corresponding channel. Each trial of the experiment was accompanied by a tactile cue of one of the above-mentioned intensities, and the time of the subject’s response to the results of each channel was recorded.

**Figure 4:**
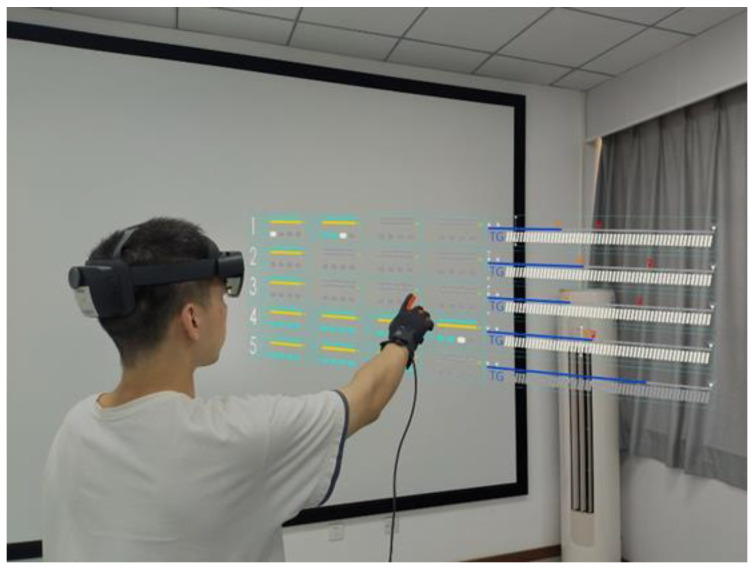
The scene of mixed reality combination in Experiment 1.

In the second stage of this experiment, after obtaining the correspondence between tactile intensity and speed, the correspondence was used to remind the subjects of the speed corresponding to the targets with high urgency to guide them to judge. In order to create a higher cognitive load, the difference in time between all targets and the target with the highest urgency arriving at the target point was set to be no more than 5% to simulate the judgment situation when multiple process targets arrived nearly simultaneously. The specific scenario was shown in Figure 4.

There were five different dynamic process channels from top to bottom in the whole visual field environment of the subjects (as shown in the figure above), and their target point starting motion positions and decision point positions were randomly generated. At the beginning of the dynamic motion of each process, the subjects observed the motion of each channel and then selected the channel with the earliest expected time to reach the decision point, i.e., the channel with the highest urgency. The experimental group was identified by adding tactile cues plus subjects’ visual observation, and the comparison group was identified by subjects observing the time and speed data of the status bar alone.

#### 2.1.2. Experimental Procedure for Single-Target Indication

This experiment is intended to simulate the precise tracking operation for a single target channel. The PRS tactile feedback scheme mentioned in the previous section was designed specifically and then applied to the domain of precise target location and tracking. By designing different PRS-specific schemes, their specific effects on cognition and task performance are explored.

**Figure 5:**
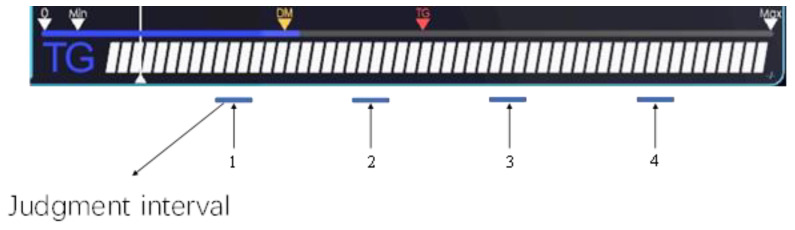
Time response point interval of single channel.

In this experimental scenario, there is a single dynamic process channel in the human visual field. In order to judge the good or bad effect of tracking and positioning more precisely, the overall area of the channel is divided into five parts. After discarding the endpoint and the starting point, there are four quintile points in the middle area. These points are taken as recording points, and a very small area (±5 cm) around the recording points is taken as the judgment interval, and when the cursor falls in the interval, the subject can both consider the state as operable, as shown in the following Figure 5.

**Figure 6:**
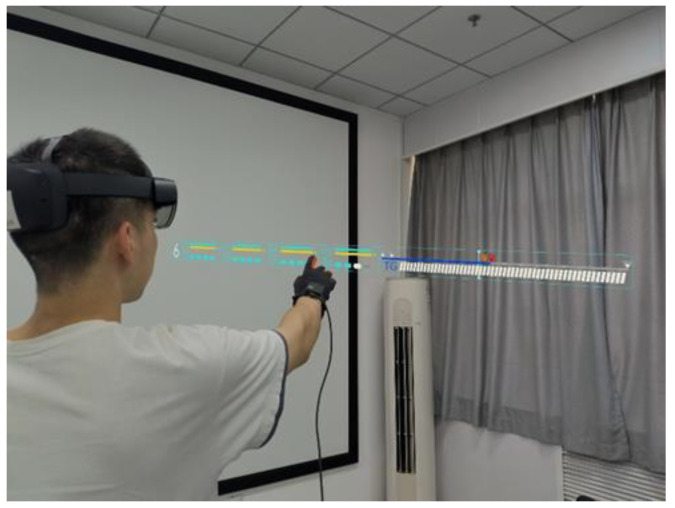
The scene of mixed reality combination in Experiment 2.

The decision was made by “clicking” in space, and the decision was considered as the completion of one trial. The target was set to move from the starting point on the left to the endpoint on the right, and its speed was varied randomly between 48 mm/s and 60 mm/s. The experimental group was aided by visual instructions with the addition of the PRS tactile scheme, and the control group was given purely visual instructions for decision-making. The following different PRS tactile protocols (listed below) were applied to the experimental groups separately for comparison experiments. The reaction time and correctness during the target movements of each scenario were recorded. The specific scenarios and visual fields were as follows (Figure 6).

Since the vibration must be position-based, the vibration position in the scheme is mandatory. Vibration rhythm and vibration sequence control the intermittency and sequence of tactile feedback, respectively, and their presence or absence has a significant impact on the design of tactile schemes for position tracking. Vibration rhythm specifically refers to a single finger in the vibration of the interval time is different, resulting in a difference in the “frequency” of human tactile perception, for example: the human body perceives the interval time of 100 ms vibration more than the 500 ms vibration to rush, but its physiological discomfort will also increase. According to existing research: when the interval time is 300 ms, the vibration effect is the best. Additionally, the vibration sequence is based on the alternation of different vibration positions, with its alternating vibration and rest to make people perceive the interval, thus promoting judgment. Therefore, the tactile indication scheme in this paper can be divided into four types as follows, where green is with this type of cue and red is without, which is shown in Table 3.

#### 2.1.3. Experimental Procedure for Comprehensive Experiment

This experiment is a process case experiment that integrates the above two experimental tasks, which is intended to simulate the interaction process of dynamic interface in the actual operation process. The effect of adding tactile cues is judged by comprehensively comparing the reaction time and accuracy rate with the case of adding tactile auxiliary indications with cases without tactile auxiliary indications.

The experimental scenario is the same as the above two tasks, but in order. After completing the urgency judgment of multiple targets in order, the participants entered the single-target judgment scene, and then carried out precise position tracking of the single target in the channel with the highest urgency, comprehensively considering the time and accuracy of the whole process: the sum of the time of the multi-objective scene judgment and the judgment time of each point of the single-objective scene tracking was used as the time criterion, and the correctness of the multi-objective judgment was used as the accuracy criterion.

### 2.2. Methods

#### 2.2.1. Method of the Principle of Multi-Objective Importance Ranking

The literature shows that there is a limit to the number of targets with different speeds that a human can perceive simultaneously [31], and the dynamic processes of multiple targets are usually accompanied by their corresponding different speeds and starting endpoints, which will cause greater difficulty for operators. In this task, there are five dynamic process channels in the same time domain. The operator needs to judge the expected time when the target reaches the destination, and then make decisions. Therefore, this paper introduces the concept of urgency to describe the relative values of the five expected times mentioned above [32]. 

The above expected time is calculated as follows:

(1) $$t=\frac{d}{v_{1}+v_{2}}$$

In describing the degree of urgency, first define the degree of urgency after the expected time $t_{i}$ and $t_{j}$, and the two channels i and j as:

(2) $$X_{i},X_{j}$$

When it is satisfied with [32]:

(3) $$\forall m\in \left[1,M\right],f_{m}(X_{i})\leq f_{m}(X_{j})\wedge \exists m\in \left[1,M\right],f_{m}(X_{i})<f_{m}(X_{j}).$$

Among them,

(4) $$f(X_{i})=t_{i}$$

Then, the degree of urgency defined as $X_{i}$ is lower than $\;X_{j}$, i.e.:

(5) $$X_{i}<X_{j}$$

In this case, the *j* channel is judged before the *i* channel, and the most urgent channel in terms of time judgment is obtained by using this method, which is the channel that should be prompted first.

#### 2.2.2. Method of Tactile Realization Principle and Tactile Indication Scheme

The tactile channel is a unique sensory channel that enables humans to acquire and perceive information by stimulating contact with the skin surface, and it does not rely on a location in space to perceive signals, so it is very useful and convenient for cues and warnings. Tactile cues are also comparable to vision and hearing in their ability to discriminate motion [33], and its cognitive mechanisms do not interfere with visual interaction [34], so it can be used as an aid to vision to achieve better results.

(1) Tactile realization principle

The parameters of tactile vibration that act on the human body include vibration intensity (*I*), vibration sequence (*S*), vibration rhythm (*R*), and vibration position (*P*), which generally characterize the sense of touch. The tactile sensation felt by the human body is mainly in the form of force stimulation [14], and for the tactile feedback glove used in this paper, the force feedback of the vibration relies mainly on the electrical pulse. This is achieved by means of stimulation of the knuckles through the set of simulated tactile electrical sensors. Setting *D* as the width of the electrical pulse and *P* as the force pressure, the linear proportional relationship is as follows [35]:

(6) $$D_{i}=\left\{ \begin{array}{cc} 0 & P<P_{\min}\\ \frac{D_{\max}-D_{\min}}{P_{\max}-P_{\min}}\times (P-P_{\min}) & P_{\min}\leq P<P_{\max}\\ D_{\max} & P\geq P_{\max} \end{array}\right.$$

The vibration rhythm is the rhythm of switching between the vibration of the pulse and the stopping vibration. In this paper, the presence or absence of the pulse vibration is expressed by 0 and 1, which constitutes a unit step function as follows. The function as the coefficient of pulse, can express the presence or absence of pulse in unit time. Its 0, 1 alternating relationship also constitutes the vibration rhythm:

(7) $$K_{d}=\left\{ \begin{array}{l} \;0\;t\;\in A\\ \;1\;t\;\notin A \end{array}\right.$$

where *A* is the time region of the vibration.

Combining the above equations and related signal theories, the tactile intensity for different rhythms and pulse pressures can be analogized to the modulation area of stimulus sensation [35]:

(8) $$K=K_{d}\int_{0}^{t}Ddt$$

**Figure 7:**
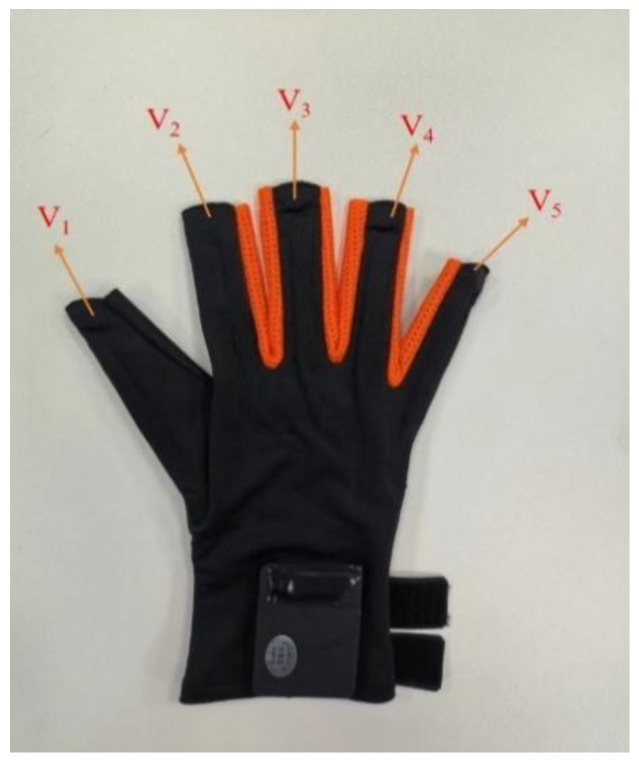
Schematic of the glove sensor.

When the time is short, the above equation can be used to describe the intensity of the vibration at the moment. The above principle is aimed at the same position of intensity and stimulation changes. The sequence of vibration is closely related to the vibration position, and when the vibration unit works differently at each different vibration position, it needs to be described and arranged according to certain rules. The five vibration locations of the tactile sensing device in this paper are located at the five knuckles and are shown in the following Figure 7. where the five contacts from the thumb to the little finger are *V*_1_, *V*_2_, *V*_3_, *V*_4_, and *V*_5_, which constitute the set of optional locations (*p*) for the entire tactile process:

(9) $$V=\left\{V_{1},V_{2},V_{3},V_{4},V_{5}\right\}$$

When the tactile vibration starts, only one or two contacts vibrate simultaneously in its position set:

(10) $$v=\left\{ \begin{array}{cc} \left\{V_{i}\right\} & D_{i}>0\\ 0 & {\;D}_{i}=0 \end{array}\right.$$

Different v-values, i.e., different vibration states, are represented, and multiple state quantities are superimposed to form a scheme of vibration sequence.

Considering that the force-pressure *p* in the above equation is not easy to measure quantitatively, the following expression is used: after receiving a stimulus, the finger will be bent by a corresponding *θ* angle, which characterizes the degree of stimulation. The feedback value measured by the tactile feedback glove in this paper is the bending angle measured by the glove’s own sensor, and then the analog signal is converted into a digital signal. The principle is: firstly, the simulated values $A_{0}$ and $\;A_{max}$, corresponding to the finger joint in bending 0 degrees and the maximum angle $\theta_{max}$ degrees are determined by initial calibration, then the simulated value $A_{\theta }$ of finger bending *θ* under the current intensity stimulus is added:

(11) $$A_{\theta }=\frac{\theta }{\theta_{\max}}(A_{\max}-A_{0})+A_{0}$$

**Figure 8:**
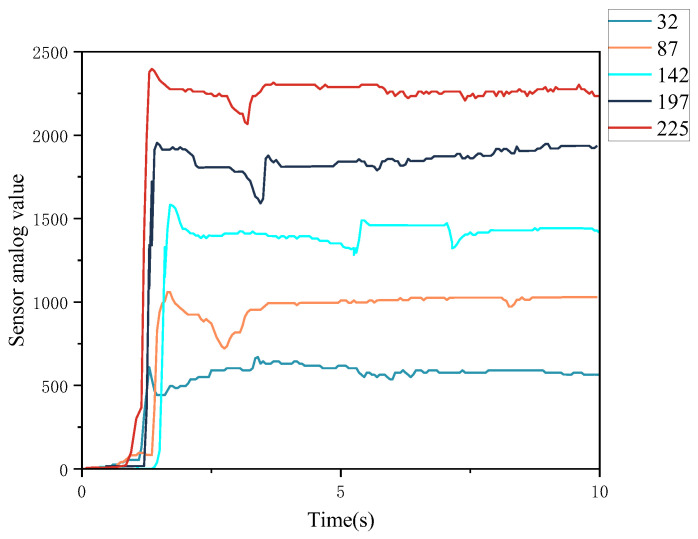
Relationship between tactile stimulus intensity and sensor feedback value.

In this paper, considering that the subjective tolerance to different objective stimulus values is not very different and the intensity is significantly positively correlated with the stimulated value, the simulated value at the time of the actual stimulus was chosen as the basis for the intensity difference in order to facilitate the quantitative study of the auxiliary indication of intensity and speed perception in the MR environment. The sensor data obtained from the tactile feedback device was analyzed for different vibration intensities, and the following feedback graph is of sensor values for the thumb over a period of time, receiving different intensities of stimuli as described in Figure 8.

As can be seen from the above figure, when the greater the intensity of vibration, the greater the simulated value of the glove sensor, which also represents the greater the degree of force proceeding, after a period of instability, the simulated value can be stabilized to refer to different intensity effects, so the use of sensor simulation value way to measure the intensity of vibration is feasible. In this paper, we will also continue to select 32, 87, 142, 197, 225 as different values of intensity differentiation, representing low, lower, medium, stronger, and strong vibration stimulation, respectively.

(2) tactile indication scheme

**Figure 9:**
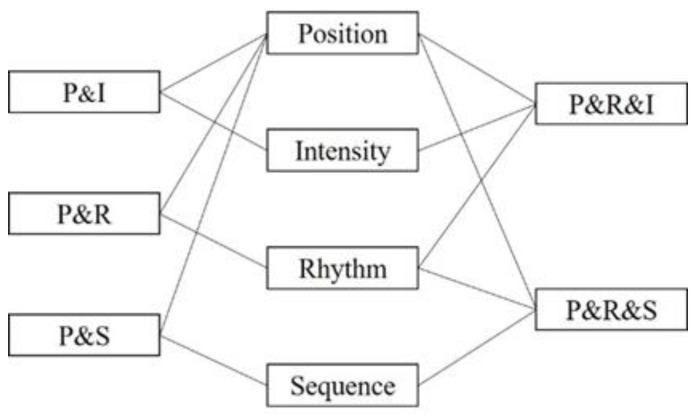
Schematic Diagram of Tactile Stimulation Scheme Combination.

The parameters of the above tactile stimuli are combined with each other to form the tactile indication method and the related scheme is shown in Figure 9 [23]:

Various types of tactile-assisted instruction schemes were used to assist operators in navigation, and other visual quick decisions.Ref. [22] showed that the PRS mode with integrated consideration of location, sequence, and rhythm has relatively the lowest impact on human cognitive load with complete cueing information, and can significantly reduce the amount of human memory. Therefore, the effect of this tactile mode on the effect of motion dynamics indication under the MR visual aid cue is not fine enough, and the specific coding scheme is not given. Therefore, experiments need to be designed to determine the mechanism of the effect of the tactile-assisted encoding scheme on visual cognition and to verify its application.

## 3. Data Analysis and Results

### 3.1. Data Analysis of Multi-Target Indication

**Figure 10:**
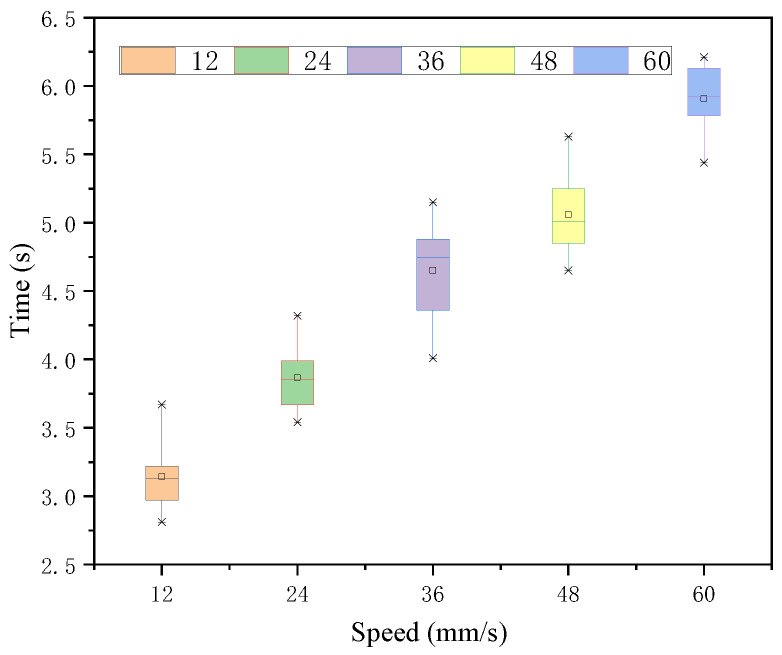
Response time of each speed target under intensity 32 stimulation.

This section is divided into two parts, the correlation analysis of the tactile intensity-speed correspondence and the comparative analysis of whether this correspondence is applied to the degree of urgency judgment. The results are shown below. In the case of each speed judgment under the 32-intensity stimulus, using within-subject one-way ANOVA, it was shown that there were significant differences in the judgment effects of each speed target under this intensity stimulus, and the results are shown in Table 4. As seen in the above Figure 10, the 12 mm/s target took the shortest time and was judged the fastest. Additionally, the judgment time speed of all five-speed targets increased in an increasing trend. The variances of their time means were tested to be not significantly different.

**Figure 11:**
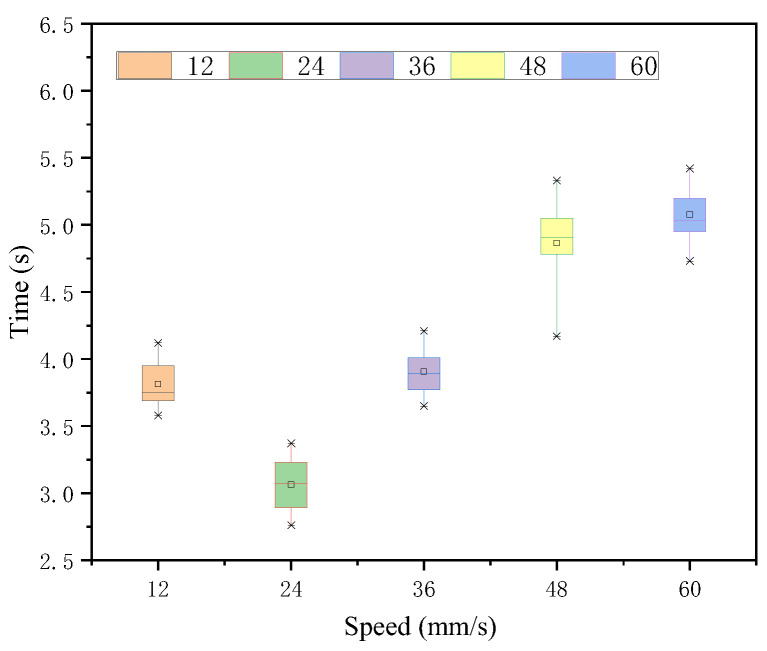
Response time of each speed target under intensity 87 stimulation.

In the case of each speed judgment under the 87-intensity stimulus, the Greenhouse-Geisser method of analyzing the temporal averages illustrates that there is a significant difference in the judgment effect of each speed target under this intensity stimulus. As seen in Figure 11 and Table 5, 24 mm/s was judged to be the fastest. The time means of 12 mm/s and 36 mm/s, which had the smallest and equal difference with 24 mm/s, were slightly larger than the time of 24 mm/s and their time means were close to no significant difference, while the response times of 48 and 60, which had a larger difference, continued to increase linearly. The variance of their time means was tested to be not significantly different.

**Figure 12:**
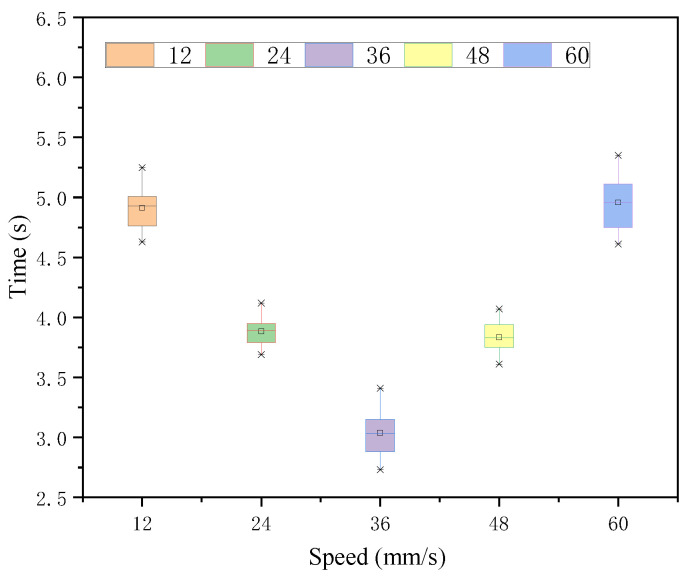
Response time of each speed target under intensity 142 stimulation.

In the case of each velocity judgment under 142 intensity stimuli, the Greenhouse-Geisser method illustrates a significant difference in the judgment effect of each velocity target under this intensity stimulus. As seen in Figure 12 and Table 6, 36 mm/s was judged the fastest. The time means of 24 mm/s and 48 mm/s, which had the smallest and equal difference with 36 mm/s, were slightly larger than those of 24 mm/s and their time means were close to no significant difference, while the response time means of 48 mm/s and 60 mm/s, which had a larger difference, were larger, and both were similar and not significantly different. The variance of their time means was tested for no significant difference.

**Figure 13:**
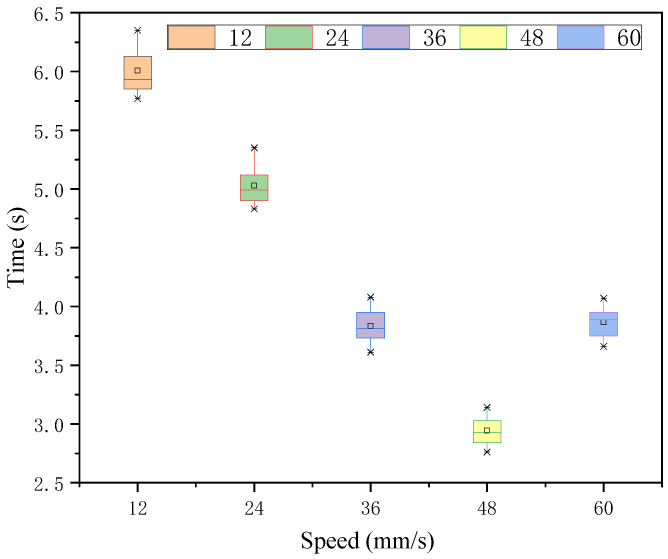
Response time of each speed target under intensity 197 stimulation.

In the case of each speed judgment under 197 intensity stimuli, the Greenhouse-Geisser method of analyzing the time means illustrates that there is a significant difference in the effect of each speed target under this intensity stimulus. As seen in Figure 13 and Table 7, 48 mm/s was judged to be the fastest. The time means of 36 mm/s and 60 mm/s, which had the smallest and equal difference with 48 mm/s, were slightly larger than those of 48 mm/s and their time means were close to each other with no significant difference, while the response time means of 12 mm/s and 24 mm/s, which had a larger difference, were also similar and had no significant difference. The variance of their time means was tested for no significant difference.

**Figure 14:**
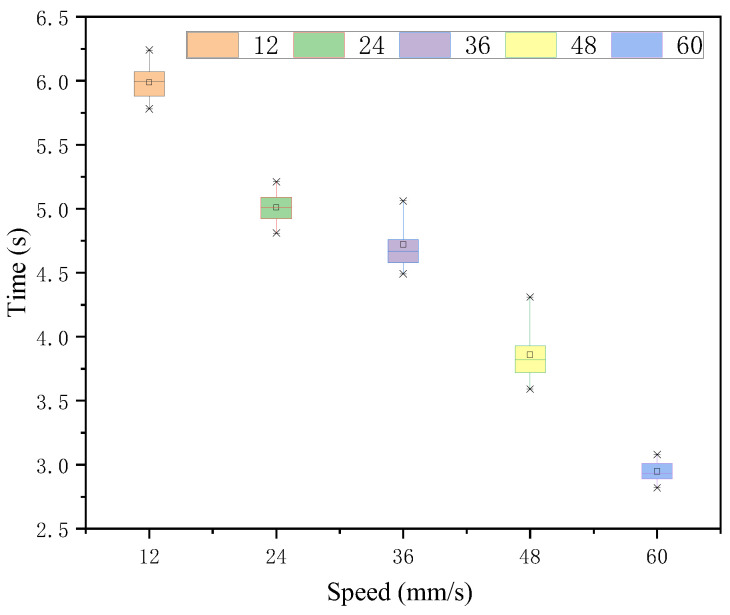
Response time of each speed target under intensity 252 stimulation.

In the case of each speed judgment under 252 intensity stimuli, the Greenhouse-Geisser method of analyzing the time means illustrates that there is a significant difference in the effect of each speed target under this intensity stimulus. As seen in Figure 14 and Table 8, 60 mm/s was judged to be the fastest. The time means of 36 mm/s and 60 mm/s, which had the smallest and equal difference with 48 mm/s, were slightly larger than those of 48 mm/s and their time means were close to each other with no significant difference, while the response time means of 12 mm/s and 24 mm/s, which had a larger difference, were also similar and had no significant difference. The variance of their time means was tested for no significant difference.

In summary, the tactile intensity-assisted visual indication of motion speed works best when the degree of stimulus intensity is matched to the speed indication in the following Table 9, and when the gap between target speed and tactile intensity and correspondence is larger, the indication is less effective, and subjects need to pay extra cognitive attention to correct the bias.

Additionally, the deviation of the target speed indication is larger for lower intensity (low, lower, medium) stimulus indication, and the error range of the matching indication is not significantly optimized, while the deviation of the target speed indication is smaller for higher intensity (higher, high) stimulus indication, and its optimization effect on the matching indication is more obvious, and the directionality of the indication is more clear and precise.

**Figure 15:**
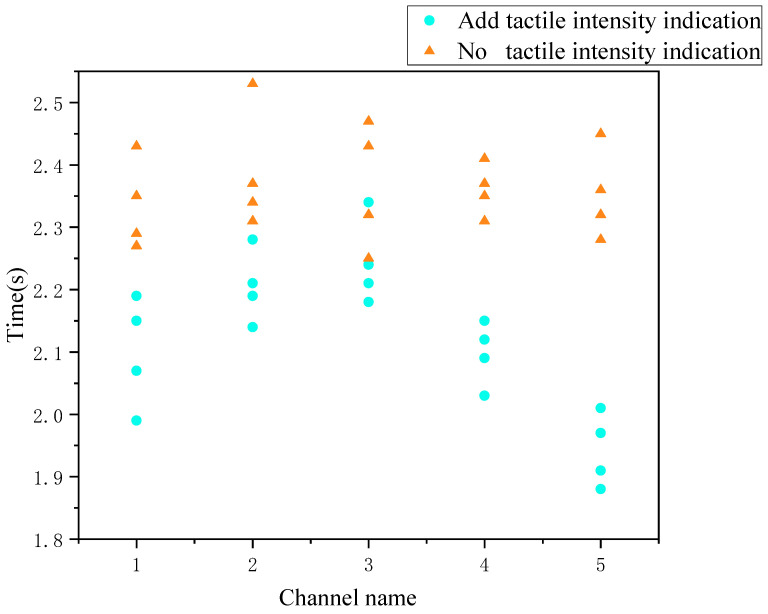
Time comparison with and without tactile intensity stimulation.

Whether to add tactile stimuli to judge the temporal distribution of urgency is such as Figure 15. 

As seen in the above figure, in general, adding tactile intensity instead of speed can significantly reduce the response decision time. An independent samples t-test for the two groups of data with and without instructions yielded that the response time of subjects who added tactile intensity cues (*M* = 2.12, *SD* = 0.12) was significantly lower than that of subjects who did not add intensity cues (*M* = 2.36, *SD* = 0.07), *t*(38) = 7.62, *p* = 0.00 < 0.05, *d* = 2.41, the difference in time means was 11.48%. A one-way between subjects ANOVA was performed on the data from the two groups of different channels with and without the addition of tactile intensity cues: *p* = 0.915 > 0.000 for the data group without the addition of tactile cues, indicating no significant difference. It can be seen that there is no difference in target discrimination between channels of different speeds with visual cues alone. While the data group with added tactile cues had a significant *p* = 0.00 < 0.05 between groups, which shows that there is a significant difference when cueing the speed channel with different tactile intensities, and according to the within-group description of the group with added cues as shown in the following Table 10.

As can be seen, channels 1 and 5, as the boundary values of the corresponding intensity cues, have a clearer effect on the subjects. Subjectively, the subjects were able to identify them better. The third channel is the middle value of the tactile vibration, and the subjects’ perception of it is somewhat ambiguous. This led to a less obvious optimization effect in this channel. The channel 5 and channel 1 time mean is 15.46%, 6.25% lower than channel 3 time with median intensity. Channels 2 and 4, as the transition channel between the boundary value and the intermediate value of the vibration intensity, also had a response efficiency between the two. The response rate of the subjects was faster when the tactile vibration intensity was higher (channels 4 and 5) than when the vibration intensity was lower (channels 1, 2, and 3). Overall, the addition of tactile intensity stimuli aided subjects well in the operation and perception of the urgency judgment task.

### 3.2. Data Analysis of Single Target Indication

**Figure 16:**
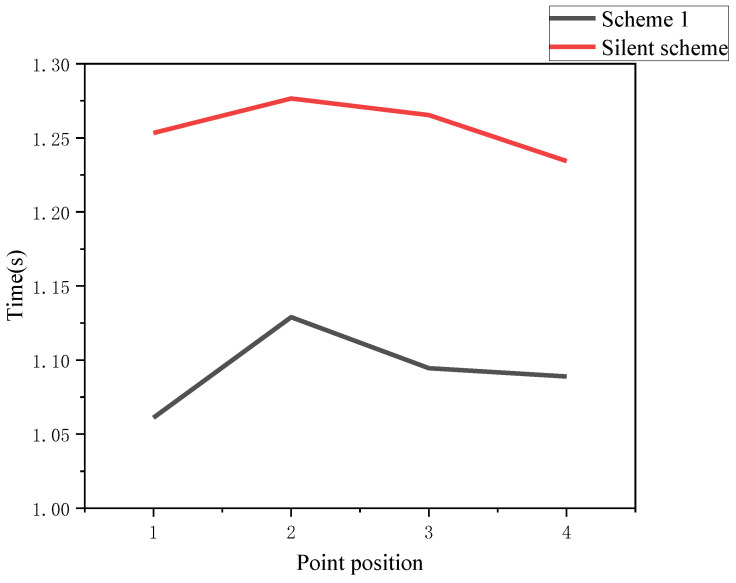
Time comparison between scheme 1 and the non-tactile scheme.

This section specifically analyzes the impact of tactile auxiliary indication scheme on subjects. First, compare the tactile scheme 1 with the non-tactile scheme in Figure 16.

**Figure 17:**
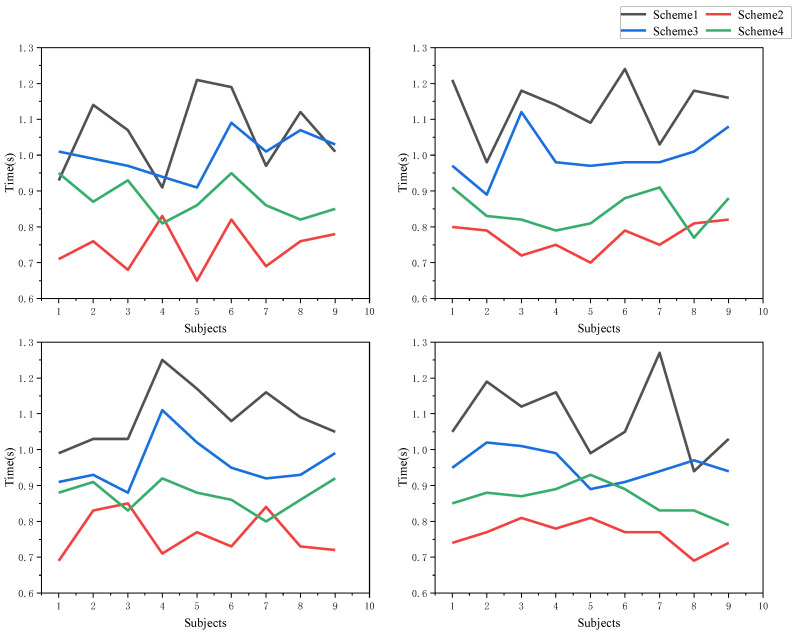
Time comparison of different schemes at each point.

It can be seen that after adding the location tactile aid cue, the subjects’ reaction time was significantly reduced, with the magnitude reaching 18.12%, 13.08%, 15.61%, and 13.36% at each point, respectively, which shows that the basic tactile cue has a better optimization effect for the location tracking task. The following analysis shows the degree of optimization of each scenario after adding the tactile cue, and the values of reaction time under each scenario are shown as Figure 17:

The raw data under the different indication schemes for each subject were subjected to significance analysis: adjusted F values obtained after one-way ANOVA for the response times at the four-time monitoring points for each subject of Scheme 1 F(2.47,7.40) = 0.299, *p* = 0.791 > 0.05, adjusted F values for each subject in Scheme 2 F(1.65,4.94) = 0.430, *p* = 0.640 > 0.05, adjusted F values for each subject in Scheme 3 F(2.19,6.56) = 1.000, *p* = 0.425 > 0.05, adjusted F values for each subject in Scheme 4 F(2.59,7.73) = 0.507, *p* = 0.664 > 0.05. It indicates that there is no significant difference in judgment time between individual subjects, and that the variability of different people can be disregarded when conducting mechanistic studies, and only the cognitive characteristics of people as a group can be considered.

The temporal data obtained above were integrated, and the 36 data indicated by the same program were divided into four groups from Program 1 to Program 4 as a group, and their data are shown in the following Table 11.

**Figure 18:**
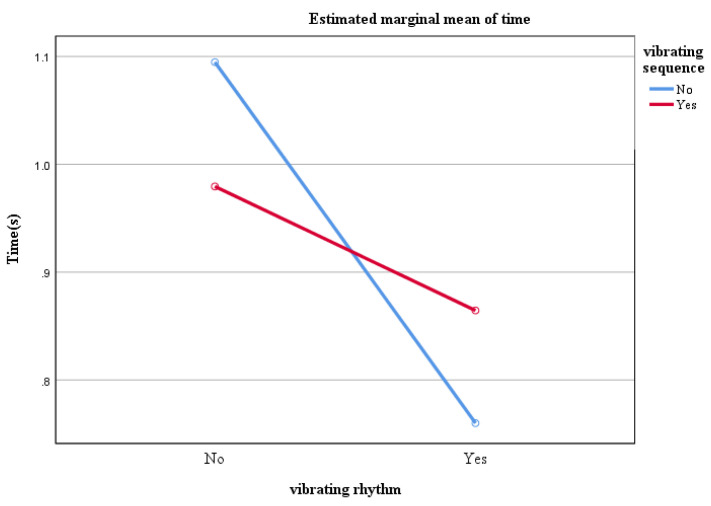
Cross Statistical Analysis of Vibration Rhythm and Vibration Stimulation.

Following the scenario design in the methodology in Section 2, the presence and absence of vibration rhythm and the presence and absence of vibration sequence were used as statistical elements to distinguish the four scenarios, i.e., scenario 1 was no rhythm and no sequence, scenario 2 was rhythm and no sequence, scenario 3 was no rhythm and sequence, and scenario 4 was rhythm and sequence, provided that the vibration location was always present. For the presence and absence of rhythm and the presence and absence of sequence, a between-subjects two-factor ANOVA was performed on the temporal data for each scenario: where the dependent variable was response time, and the independent variables were vibration rhythm (presence and absence) and vibration sequence (presence and absence). The results showed that the main effect of vibration rhythm was significant, F(1,140) = 406.19, *p* = 0.00 < 0.05,$\;\eta^{2}=0.74$, according to the evaluation criteria given by Cohen [36], the effect value is large where the reaction time of the cueing scheme with vibrational rhythms involved was significantly smaller than that without rhythms. The main effect of vibrational sequence is not significant, F(1,140) = 0.24, *p* = 0.628 > 0.05,$\;\eta^{2}=0.002$, the effect value is small. The response time with the vibration sequence cue was slightly smaller than that without the cue but the difference was not significant. The interaction of rhythm and sequence is significant, F(1,20) = 96.96, *p* = 0.00 < 0.05,$\;\eta^{2}=0.41$, the effect value is large. For the overall cue, the vibration rhythm can improve the response efficiency with or without the vibration sequence, but the improvement effect is better when there is no sequence; while in the premise of the vibration rhythm, adding the vibration sequence cue will reduce the response efficiency, which reflects that too many tactile cue elements will cause redundancy to human cognition as shown in Figure 18.

Additionally, through the above analysis, after excluding human subjective differences, in order to reduce the impact of a single experiment on the overall error, we summarized the time data of all subjects at each point according to the following mean value principle, where E is the calculated experimental data, $E_{i}$ the time value of the subject *i*, and *n* is the number of subjects:

(12) $$\bar{E}=\frac{\sum_{i=1}^{n}E_{i}}{n}$$

**Figure 19:**
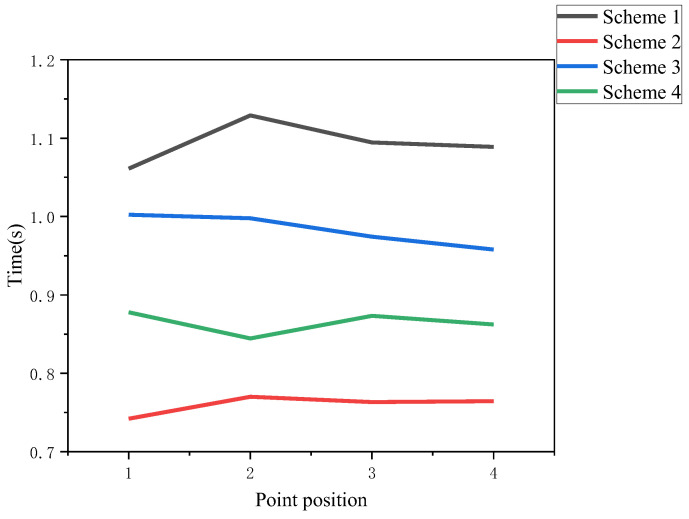
Mean value of different schemes at each point.

Additionally, the time mean distribution of different schemes at each point is plotted as shown in Figure 19:

Analyzing the above data, the time averages of scheme 2, 3, and 4 decreased by 30.49%, 10.09%, and 20.93%, respectively, compared to that of scheme 1, and scheme 2 where it is less difficult to implement and use less equipment than the more efficient scheme 3 at the same time. Therefore, scheme 2 has the best prompting effect, followed by scheme 4 and scheme 3, and scheme 1 has a slightly inferior prompting effect.

### 3.3. Data Analysis of Comprehensive Experiment

**Figure 20:**
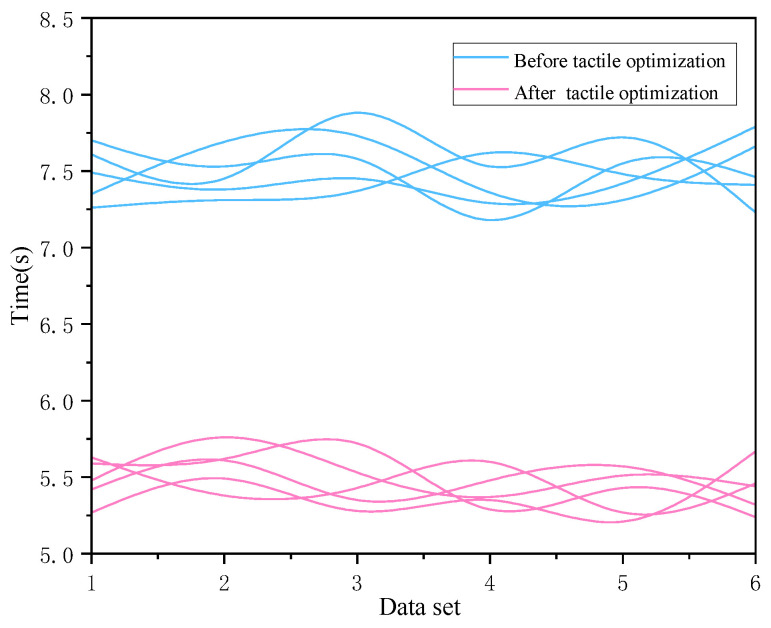
Comparison chart of comprehensive experiment time.

The time distribution of the comprehensive experiment is shown in the Figure 20 below:

Analysis of variance was performed on the time data before and after the above optimization, the reaction time of the experimental group with a tactile cue (*M* = 5.45, *SD* = 0.15) was significantly lower than that of the experimental group without a tactile cue (*M* = 7.49, *SD* = 0.18), *t*(29) = 54.88, *p* = 0.000 < 0.05, *d* = 10.02, This effect data is large.

This task requires a combination of time and accuracy. According to the time accuracy comprehensive evaluation model of Maris Gunter [37]:

(13) $$\sum_{i}\left(C_{i}X_{pi}-P_{i}(1-X_{pi}))(d-T_{pi}\right)$$

where $X_{i}$ is the judgment performance, the value is 1 when the judgment is correct, 0 when it is wrong, $C_{i}$ is the coefficient of increase of the fraction when the reaction is correct, $P_{i}$ is the reduction factor of the fraction in case of incorrect reaction, and *d* is the time limit. This value is generally the maximum time to complete the task, $T_{pi}$ is the time to respond. Since the time of all judgments is less than 8 s, we set the time d in the formula to 8. The evaluation method of Maris Gunter was optimized into the following Equation (14) applicable to this experiment.

(14) $$\sum_{i:d_{i}=8}\left(C_{i}X_{pi}-P_{i}(1-X_{pi}))(d-T_{pi}\right)$$

where the values of $C_{i}$ and $P_{i}$, as the core part of the overall scoring method need to be set by expert opinion in the field. Here, we analyze the importance of the four experts related to the time and correctness of completing the task by adopting the AHP method [38]. Set $C_{i}$ and $P_{i}$ ratio to 4.65:1. As shown in the following formula:

(15) $$\sum_{i:d_{i}=8}\left(X_{pi}-4.65(1-X_{pi}))(d-T_{pi}\right)$$

**Figure 21:**
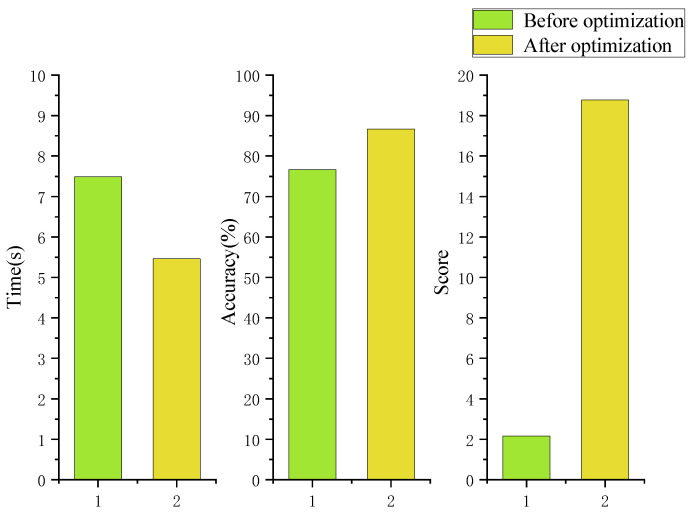
Comparison chart of experimental results.

The final correct rate and reaction time were brought into the above equation to obtain a combined performance score of 18.77 after tactile optimization and 2.16 before optimization. The combined correctness, response time, and score of the combined experiment are displayed in order in Figure 21.

## 4. Discussion and Analysis

Based on the current demand for precise and rapid judgment of motion perception tasks in the MR environment, this paper innovatively uses tactile sensors to assist human visual interaction and assists people’s multi-target urgency perception and single-target accurate tracking perception through tactile intensity prompts and different vibration scheme prompts. Through experiments in two different scenarios and a comprehensive experiment, the relationship between motion speed and tactile intensity in MR environment and its application in urgency discrimination, as well as the relationship between single-target precise positioning and different tactile indication schemes, are explored, and the effectiveness of tactile-assisted visual comprehensive indication is confirmed, the specific conclusions are as follows:

(1) Experiment 1 is an experiment on the discrimination of velocity and importance of multi-target channels in MR environment, and its purpose is to explore the application effect of the tactile intensity-velocity relationship in importance judgment. In the first stage, the coupling relationship between speed and vibration intensity is explored by setting vibration of different intensities to match the speed channel. The obtained reaction time is used as a judgment indicator. After data analysis, the *p*-value of the significance test for each data set under each intensity was less than 0.05. That is, when each intensity corresponds to a target at different speeds, the participant’s judgment time is significantly different. When the vibration intensity-target speed (sensor value-target speed) correspond to low-slow (32–12 mm/s), lower-slower (87–24 mm/s), medium-medium (142–36 mm/s), higher-faster (197–48 mm/s), and high-fast (252–60 mm/s), the judgment time is the shortest and the effect is the best. In the second stage, this paper applies this relationship to the judgment of target urgency, and through comparative experimental analysis, the mean reaction time of the experimental group with tactile intensity-velocity relationship cue is at least 11.48% lower than that of the control group without tactile intensity cue, and the difference between the groups is *p* = 0.00 < 0.05, *t*(38) = 7.62, and the effect value *d* = 2.41(large), which is a large overall difference. Moreover, under the premise of adding the tactile intensity indicator, the prompt effect is also significantly different between different channels (*p* = 0.00 < 0.05), among which the time means of channel 5 with the highest intensity and channel 1 with the smallest intensity are 15.46% and 6.25% lower than that of channel 3 with intermediate intensity. 

(2) Experiment 2 is an experiment of single-target channel target tracking in MR environment, the purpose of which is to assist the participant in obtaining the precise position of a single target and tracking through different PRS-based tactile indication schemes, so as to obtain the effectiveness of the specific tactile scheme based on PRS for visual judgment position. Firstly, the difference between scheme 1 (position) and no vibration scheme cues was compared. After adding the positional tactile assist cue, the reaction time of the participants decreased by 18.12%, 13.08%, 15.61%, and 13.36% at each point, respectively. Then, through the design experiment, the effect of each tactile indication scheme on the auxiliary vision to accurately locate the moving target was explored, and the conclusion was as follows: the time of schemes two, three, and four decreased by 30.49%, 10.09%, and 20.93%, respectively, compared with scheme 1. Therefore, the cueing effect of scheme 2 is the best, followed by scheme 4 and scheme 3. The cueing effect of scheme 1 was slightly less effective. The ANOVA is performed on the scheme group that adds rhythm, rhythm & sequence, and sequence on the basis of position. The vibration rhythm and rhythm & order of *p* = 0.00 < 0.05, the F values were 406.19 and 96.96, and the effect values $\eta^{2}$ were 0.74 and 0.41, respectively, indicating that the influence of both on the reduction of judgment time was significant, and the optimization degree of vibration rhythm was high. However, the *p*-value of the addition sequence scheme is 0.628 is greater than 0.05, the F value is 0.24, and the effect value $\eta^{2}$ is 0.002, indicating that its effect on time reduction is not significant.

(3) Experiment 3 is a comprehensive process case experiment combining the first two experimental tasks, and its purpose is to judge the effect of adding the above tactile cue optimization on the overall dynamic target perception. Through the analysis, the optimized reaction time is reduced by 27.15% compared with the pre-optimization period, and the accuracy rate is increased by 11.54%. Additionally, through the score of comprehensive time and correct rate, it is 18.77 after optimization and 2.16 before optimization, which has a large improvement and good optimization effect.

## 5. Conclusions

This paper mainly designs the tactile auxiliary visual indication method in the two cases of multi-target simultaneous perception and single-target accurate perception, and judges its optimization effect through experiments, and the conclusion is as follows:

(1) There is a tactile intensity-velocity correspondence in the MR environments: high, higher, medium, lower, and low tactile stimulus intensities induce human visual attention to be biased toward fast, faster, medium, slower, and slow motion targets, and the shortest judgment time and highest efficiency when the two are matched, and the conclusion is very effective when applied to the actual scenario of judging the urgency of the target, and the greater the degree of differentiation of intensities, the higher the efficiency of human discrimination.
(2) The PRS tactile indication scheme will better enhance the precise motion localization of a single target by distinguishing the difference between the presence and absence of vibration rhythm and vibration sequence, and the PRS theory-guided tactile scheme is specified as four schemes: position, position & rhythm, position & sequence, and position & rhythm & sequence. In general, the above schemes have good results for precise position localization of single motion targets. Among them, compared with scheme 1 that only prompts the vibration position (P), scheme 2 that adds vibration rhythm (R) and scheme 4 that adds vibration rhythm (R) and sequence (S) at the same time can greatly improve the efficiency of human judgment position, and scheme 2 has a better effect.
(3) In the process case synthesis experiment, adding tactile comprehensive indication can significantly improve the accuracy of human perception of target movement and reduce reaction time.

The above findings provide a better optimized idea and scheme design for the visual-tactile interaction in MR environment, a new idea and new method for the velocity and position judgment task of tactile-assisted vision in MR holographic environment, and a better theoretical basis for the subsequent mechanism of perception and cognition of spatial motion information.

## Figures and Tables

**Table 1:** This is a table. Tables should be placed in the main text near to the first time they are cited.

Channel	1	2	3	4	5
Speed (mm/s)	60	48	36	24	12

**Table 2:** Correspondence between sensor data and tactile intensity, Intensity level.

Sensor Data	32	87	142	197	252
Intensity(v)	0.8	1.6	2.4	3.2	4
Intensity level	low	lower	medium	higher	high

**Table 3:** Schematic diagram of tactile scheme.

	Vibration Position	Vibration Rhythm	Vibration Sequence
Scheme 1	●	●	●
Scheme 2	●	●	●
Scheme 3	●	●	●
Scheme 4	●	●	●

**Table 4:** Time statistic value under intensity 32.

	*M*	*SD*	*F*	*p*
12	3.14	0.24	105.24	0.000 < 0.05
24	3.87	0.23		
36	4.65	0.37		
48	5.06	0.29		
60	5.91	0.24		

**Table 5:** Time statistic value under intensity 87.

	*M*	*SD*	*F*	*p*
12	3.81	0.17	124.00	0.000 < 0.05
24	3.06	0.19		
36	3.90	0.18		
48	4.86	0.32		
60	5.08	0.20		

**Table 6:** Time statistic value under intensity 142.

	*M*	*SD*	*F*	*p*
12	4.91	0.19	159.90	0.000 < 0.05
24	3.88	0.13		
36	3.04	0.20		
48	3.83	0.15		
60	4.96	0.24		

**Table 7:** Time statistic value under intensity 197.

	*M*	*SD*	*F*	*p*
12	6.01	0.20	531.07	0.000 < 0.05
24	5.03	0.16		
36	3.83	0.14		
48	2.94	0.12		
60	3.87	0.13		

**Table 8:** Time statistic value under intensity 252.

	*M*	*SD*	*F*	*p*
12	5.99	0.14	485.15	0.000 < 0.05
24	5.01	0.12		
36	4.72	0.19		
48	3.86	0.21		
60	2.95	0.08		

**Table 9:** Correspondence between sensor value and target speed.

Sensor values	32	87	142	197	252
Target speed (mm/s)	12	24	36	48	60

**Table 10:** Time statistics of each channel.

Serial Number	Number of Cases	Average Value	Standard Deviation	95% Confidence Interval		Upper Limit	Maximum Value
				Lower Limit	Upper Limit		
1	4	2.10	0.08869	1.9589	2.2411	1.99	2.19
2	4	2.21	0.05802	2.1127	2.2973	2.14	2.28
3	4	2.24	0.06946	2.1320	2.3530	2.18	2.34
4	4	2.10	0.05123	2.0160	2.1790	2.03	2.15
5	4	1.94	0.05852	1.8494	2.0356	1.88	2.01

**Table 11:** Time Cross Statistical Analysis of Sequence and Rhythm.

Rhythm	Sequence	*M*	*SD*
No	No	1.09	0.096
	Yes	0.98	0.062
	Total	1.04	0.099
Yes	No	0.76	0.051
	Yes	0.86	0.047
	Total	0.81	0.072
Total	No	0.93	0.185
	Yes	0.92	0.079
	Total	0.92	0.142

## Data Availability

The data presented in this study are available on request from the corresponding author. The data are not publicly available due to some data involve detailed equipment parameters, these parameters cannot be disclosed.
